# Diagnosis of early kidney allograft rejection: influencing factors in metabolite-based urine analysis

**DOI:** 10.3389/fmed.2026.1688235

**Published:** 2026-03-06

**Authors:** Katharina Wiesner, Eric Schiffer, Franz Josef Putz, Andrew Robertson, Simone Mark, Simone Reichelt-Wurm, Katharina M. Schmidt, Amauri Schwaeble Santamaría, Bernhard Banas, Miriam C. Banas

**Affiliations:** 1 Department of Nephrology, University Hospital Regensburg, Regensburg, Germany; 2 Department of Internal Medicine and Geriatrics, Krankenhaus Barmherzige Bruder Regensburg, Regensburg, Germany; 3 numares AG, Regensburg, Germany; 4 Department of Surgery, University Hospital Regensburg, Regensburg, Germany; 5 Institute for Experimental Molecular Imaging, Rheinisch-Westfalische Technische Hochschule Aachen, Aachen, Germany

**Keywords:** allograft rejection, biopsies, kidney transplantation, metabolomics, urine test

## Abstract

Acute allograft rejection remains a major complication in kidney transplantation, highlighting the need for accurate and early diagnostic tools to enable prompt treatment. Standard diagnostic methods, such as serum creatinine monitoring, lack sufficient sensitivity and specificity. As a result, subclinical rejection may go undetected, or unnecessary biopsies may be performed, posing additional risks. Previously, a novel, non-invasive urine test based on metabolomic profiling was developed to detect renal allograft rejection. However, the early post-transplant period remains particularly challenging, as reliable biomarker-based detection in this critical window is not yet well established. This study, conducted within the UMBRELLA project, analyzed 682 urine samples from 109 kidney transplant recipients. The test utilized a specific urinary metabolite constellation based on alanine, citrate, lactate, and urea. A total of 29 clinical and transplant-related parameters, including donor and recipient characteristics, ischemia times, and donor type, were evaluated for their effect on the test’s ability to detect biopsy-confirmed rejection within the first 14 days after transplantation. Univariate analysis identified 10 significant confounding factors, including lower residual urine volume before transplantation, reduced eGFR, use of induction therapy, longer warm and cold ischemia times, deceased donor status, younger recipient age, and certain HLA mismatches. Multivariate analysis confirmed the relevance of living donation. Subgroup analysis revealed the highest diagnostic accuracy in recipients of living donor kidneys, with an AUC of 0.720 (95% CI, 0.62–0.82), followed by recipients with a short warm ischemia time (<30 min), who achieved an AUC of 0.702 (95% CI, 0.61–0.79). Clinical complications often coincided with abnormal metabolite test results. In conclusion, this study underscores the importance of considering donor type and ischemia times when interpreting urinary metabolite constellations for rejection monitoring in the early post-transplant period. The findings suggest a distinct metabolic profile in recipients of deceased donor kidneys within the first 2 weeks after transplantation. Understanding these influencing factors may enhance the accuracy of non-invasive rejection detection and support timely clinical interventions to improve patient outcomes.

## Introduction

Kidney transplantation, whether from a living or deceased donor, is the preferred treatment for patients with end-stage chronic kidney disease. This is primarily due to higher survival rates and improved quality of life for transplant recipients compared to those undergoing dialysis (1, 2).

One of the major complications following renal transplantation is acute allograft rejection, which occurs in 5.1–8.0% of kidney transplant recipients within the first year (3). Advances in treatment options over the past decades have improved outcomes for patients experiencing rejection (4). However, the success of these treatments relies on early and accurate diagnosis.

According to KDIGO guidelines, the standard method for monitoring kidney transplant recipients is serum creatinine measurement and estimation of glomerular filtration rate (GFR). An increase in serum creatinine levels prompts a renal transplant biopsy, which is considered to be the gold standard for diagnosing renal allograft rejection (5). However, this procedure is invasive and carries risks such as bleeding, limiting its use for continuous monitoring (6). Additionally, elevated serum creatinine levels are not specific for allograft rejection, leading to a significant number of unnecessary biopsies. Furthermore, serum creatinine lacks sensitivity in detecting subclinical allograft rejection—a condition in which patients show no overt signs of rejection but may still benefit from early intervention with anti-rejection therapy to improve long-term outcomes (7, 8).

Given these limitations, there is an urgent need for a non-invasive, cost-effective, and reliable method to monitor renal allografts for rejection. Metabolomics has emerged as a promising approach in this field (9). Banas et al. developed a non-invasive urine test that detects acute renal allograft rejection based on a specific constellation of urine metabolites: alanine, citrate, lactate, and urea (10). These metabolites have been previously reported as altered in renal dysfunction (11–14).

Following its initial discovery, this constellation demonstrated clinical relevance in the prospective, observational UMBRELLA study, achieving an area under the receiver operating characteristic curve (AUC) of 0.75 for detecting renal rejection (15). However, the test was only effective when applied to urine samples collected from day 15 post-transplant onward. In contrast, during the first 2 weeks post-transplant, the metabolite constellation showed no diagnostic significance (AUC of 0.52).

Challenges in detecting kidney rejection in the early post-transplant period are not unique to metabolomics. For instance, donor-derived cell-free DNA (ddcfDNA) levels take at least 10 days to stabilize (16), and within the first 3 months after transplantation, ddcfDNA has not been shown to significantly outperform serum creatinine in detecting rejection (17). Similarly, Torque Teno virus (TTV) levels require approximately 3 months to reach a steady state before becoming useful as a tool to detect kidney rejection (18, 19).

These observations formed the basis for this post-hoc analysis of the UMBRELLA cohort (15). The aim was to identify potential confounding factors affecting urine metabolite profiles within the first 14 days post-transplant. Additionally, we sought to define a specific subgroup in which the metabolomics-based urine test could be applicable during this early period.

## Materials and methods

### Study design

As part of the prospective, observational UMBRELLA study, a total of 109 participants were recruited at the University Hospital Regensburg between 2011 and 2012. The study protocol has been described elsewhere (15). Briefly, patients received either a kidney transplant alone (*n =* 100) or a combined kidney and pancreas transplant (*n =* 9). Enrollment began on the day of transplantation (day 0) and included 1 year of follow-up. The analysis presented here focuses on the first 14 days post-transplant, referred to as the “inpatient” phase, regardless of individual discharge dates.

**Figure 1:**
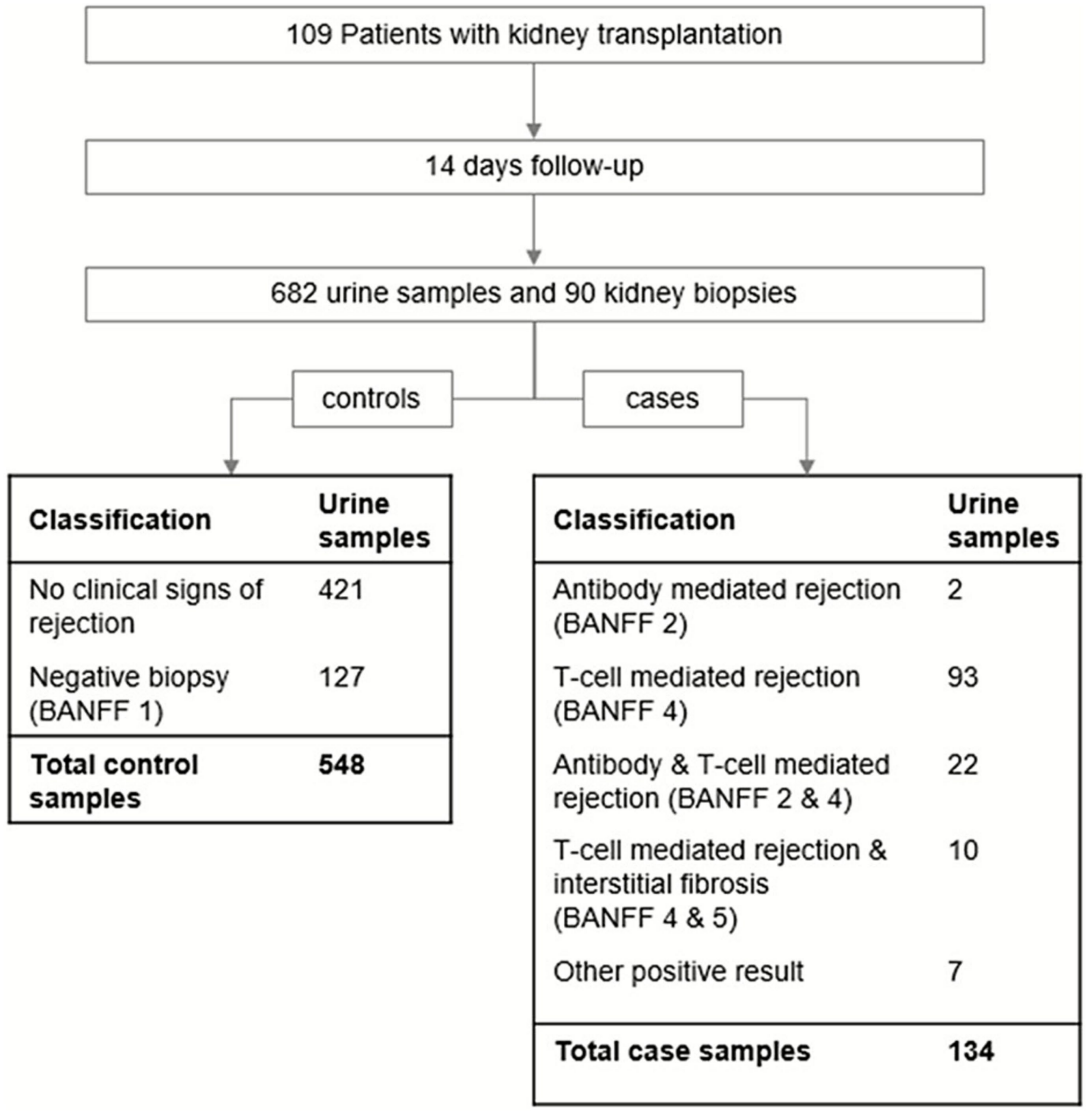
Study design and sampling flow-chart. A total of 109 patients with kidney transplantation alone (*n =* 100) or combined kidney pancreas transplantation (*n =* 9) were followed from the day of transplantation (day 0) to day 14. Urine samples were usually taken daily and processed according to study protocol (15). A total of 682 urine samples finally were available for analysis passing all quality controls and having a clear allocation. During the first 14 days after transplantation, 90 kidney biopsies were performed and categorized according to the then-current BANFF criteria. In the event of histopathological findings indicating kidney rejection, the respective biopsy was classified as a case. ‘Other positive results’ were obtained for histopathological findings that were clearly identified as acute rejection but without the possibility to allocate them according to BANFF criteria. Aligning the result of the biopsy to the urine samples which were taken in a 7 day window before the biopsy, it resulted in 548 control and 134 case samples.

Urine samples were intended to be collected daily during the inpatient phase and analyzed using nuclear magnetic resonance (NMR) spectroscopy. Sample preparation and assignment followed exactly the protocol as previously described (15). As part of the center’s standard protocol, a kidney biopsy was performed on day 14 ± 2 prior to discharge. In total, 90 biopsies were performed within the first 14 days and histopathologically evaluated according to the then-current BANFF classification (20). All urine samples collected during an uneventful clinical course or before and after a negative biopsy were considered controls. Samples collected within 7 days prior to or on the day of a biopsy with histopathologically confirmed rejection were classified as cases. Samples taken after the initiation of a rejection treatment or alongside a biopsy with an unclear finding were excluded. This resulted in a total of 682 eligible urine samples during the inpatient phase, including 548 control samples and 134 case samples (see Figure 1).

### Clinical data preparation

All clinical data collected were reviewed, and 32 potential confounding factors were selected for analysis. Some factors required data preprocessing, such as grouping free-text fields related to the initial disease. Three parameters were excluded due to insufficient sample numbers: BKV infection (*n =* 0), BK nephropathy (*n =* 1), and panel reactive antibodies (*n =* 10) (Supplementary Table 1).

For 78 samples, no estimated glomerular filtration rate (eGFR) value was available on the day of urine collection. If eGFR data were available for the previous or following day, that value was used. This resulted in 50 samples lacking eGFR data.

Additionally, individual urine rejection score plots were generated for all patients over the first 20 days. These plots were visually inspected, and abnormalities—such as high rejection scores in the absence of clinical signs or evidence of rejection—were noted. Any clinical irregularities during these episodes, including surgical or postoperative complications, were also documented.

### Statistical analysis

All statistical analyses were performed using R software (version 3.5.3), and results are reported in accordance with STARD guidelines.

The performance of the previously developed metabolite rejection score in discriminating urine samples from patients with acute rejection versus those without was assessed using the area under the receiver operating characteristic curve (AUC). ROC plots were generated for both the full dataset and the predefined subgroups, including 95% confidence intervals (CIs) calculated via 2,000 stratified bootstrap replicates. AUC values and their CIs were used to evaluate performance.

AUC calculations and pairwise comparisons were conducted using the pROC v1.15.3 and cvAUC v1.1.0 packages, for pooled repeated measures data. Correlation coefficients were computed using Pearson’s correlation (cor from the stats package).

Univariate analyses were performed separately for cases and controls across three different cutoff settings according to previously published data (15): a highly sensitive cutoff (score = 3), a highly specific cutoff (score = 13), and a mixed cutoff where samples with scores between 3 and 13 were excluded. This resulted in six analysis scenarios aimed at identifying parameters with significant differences between correctly and incorrectly classified samples (*α* = 0.05).

The Mann–Whitney U test was employed for continuous variables, while Pearson’s chi-square test was used for categorical factors. In both cases, *p*-values were adjusted using the Bonferroni correction. Statistical significance was defined as an adjusted *p* < 0.05. The chi-square test was considered invalid if the expected value in at least one cell was less than five.

Multivariate analysis was performed using an exhaustive search approach within a previously established multiple logistic regression model for metabolite score detection (10). All possible combinations of two parameters were evaluated. For each parameter set, a logistic regression model was fitted to predict the classification (true/false) of each sample. The results of the exhaustive search included a list of candidate models along with their corresponding AUC values. These AUC values were used to filter models based on their performance. Pairwise AUC comparisons between biomarker combinations were performed using DeLong’s nonparametric test, which tests the equality of AUCs.

## Results

In order to better understand the factors influencing test performance in the early post-transplant period, we performed a detailed post-hoc analysis of clinical and metabolomic data from the UMBRELLA study.

### Univariate analysis

The clinical data collection identified 29 potential confounders (Supplementary Table 1). As an initial step in the statistical analysis, univariate statistics were performed separately for cases and controls using three different cut-offs: 3 (for high sensitivity), 13 (for high specificity), and a setting in which samples with scores between 3 and 13 were excluded. Each parameter was analyzed under six different settings to identify those that significantly (*α* < 0.05) differentiated between correctly and incorrectly classified samples. Overall, 10 of the 29 parameters showed significant associations with score classification in at least one setting (Table 1).

**Table 1:** Overview of the results of the univariate analyses.

Number of significant settings	Parameter	Controls, cut-off 3	Controls, cut-off 13	Controls, mixed cut-off	Cases, cut-off 13
3	Induction therapy	0.0571 = n.s.	0.0002	0.0067	0.0144
3	HLA-DR mismatch	<0.0001	0.0063	<0.0001	n.s.
3	Warm ischemia time	0.0330	0.0070	0.0001	n.s.
3	eGFR	0.0263	0.027	0.0020	n.s.
2	Residual excretion	n.s.	0.0002	0.0006	n.s.
2	Cold ischemia time	n.s.	0.0015	0.0017	n.s.
2	Living donor	n.s.	0.0011	0.0044	n.s.
2	Age	n.s.	0.0154	0.0067	n.s.
2	Total HLA mismatch	0.0007	0.0129	E < 5* < 0.001	n.s.
1	HLA-A mismatch	0.0269	n.s.	n.s.	n.s.

In univariate analysis, the 29 potential confounding factors were examined using Mann–Whitney U test for continuous variables and Pearson’s chi-squared test for categorical parameters to determine whether they have a significant influence on the correctness of the urine metabolite score classification. The analyses were performed separately for case and control samples and for three different cut-off settings (highly sensitive, highly specific, and a mixed cut-off in which all samples between the two cut-offs were excluded). Bonferroni-corrected p values for multiple testing are shown in each parameter/setting combination in the respective fields. A value below 0.05 was considered significant. Of all parameters in all settings, exclusively the combinations shown here included statistically significant results. No significant results were obtained for cases with cut-off 3 and mixed cut-off, therefore they are not presented here. p values greater than or equal to 0.1 are displayed as n.s. (not significant). In the first column, the number of settings is presented in which the respective parameter was significant. *(E < 5) If the expected value in the chi-squared was below five at least in one cell, the result was not considered valid.

Parameters associated with correct classification included induction therapy, one HLA-DR mismatch, shorter warm ischemia time, higher estimated glomerular filtration rate (eGFR), higher residual excretion, shorter cold ischemia time, living donor status, older recipient age, and a total of three HLA mismatches. Three parameters— HLA-DR mismatch, warm ischemia time, and eGFR—were significant across all three control settings. In two of the three control settings, six parameters—induction therapy, residual excretion, cold ischemia time, living donor status, age, and total HLA mismatch—were relevant. The HLA-A mismatch was only significant in the high sensitivity control setting. Confounders were identified exclusively in the control setting, except for induction therapy, which also showed significance in one case setting.

### Multivariate analysis

While univariate analysis offers insights into individual parameters associated with correct classification, it does not account for potential interactions between variables. Many clinical factors are interdependent, and their combined effects may differ from their individual influences. To identify the most influential predictors while controlling for confounders, the interrelationships between parameters were examined using multivariate logistic regression models.

All parameters significant in univariate analysis, along with additional clinically relevant variables such as sex, presence of infection, or leukocyturia (see Supplementary Table 1), were included. For each pairwise combination of two parameters, the ability to distinguish correctly from incorrectly classified samples was assessed via the area under the receiver operating characteristic curve (AUC).

The results were filtered based on three criteria: (1) an AUC of at least 0.7, (2) a minimum of 30 samples in both the correctly and incorrectly classified groups, and (3) a significant improvement in classification performance compared to either parameter alone. This process identified 12 instances where the combination outperformed individual parameters.

Models remained only in two control settings, specifically, at the cut-off of 13 and the mixed cut-off. These models included exclusively seven of the 10 parameters previously identified as significant in univariate analysis. Residual excretion was the most frequently involved parameter, appearing six times as part of the combination and one time as the sole parameter, with its classification ability enhanced when combined with others. The other parameters included induction therapy, age, HLA-DR mismatch, total HLA mismatch, living donor status, and cold ischemia time. All parameter combinations are presented in Table 2.

**Table 2:** Multivariate analysis results using multiple logistic regression.

Setting	Compared parameter	Combined parameter	AUC of the combination	*p-*value (DeLong)
Controls, cut-off 13	Residual excretion	Age	0.73	0.0165
	Age	Residual excretion	0.73	0.0006
	Cold ischemia time	Residual excretion	0.71	0.0144
Controls, mixed cut-off	Induction therapy	Residual excretion	0.77	0.0222
	HLA-DR mismatch	Residual excretion	0.76	0.0026
	Total HLA mismatch	Residual excretion	0.75	0.0017
	Living donor	Residual excretion	0.73	0.0175
	Living donor	Age	0.71	0.0298

A multiple logistic regression model was used for multivariate analysis in order to calculate the interferences between parameters. This was done by exhaustively searching for all possible combinations of two parameters that best discriminate between correct and incorrect classifications. Finally, the Area Under the Curve (AUC) of the Receiver-Operating-Characteristic (ROC) curve of the combination of two parameters was compared with the AUC of every parameter alone. This table shows all combinations in which the AUC of the combination was greater than 0.70, which had at least 30 samples in each of the correctly and incorrectly classified groups and in which the combination was significantly better at distinguishing between correctly and incorrectly classified than the compared parameter alone (*p*-value DeLong < 0.05).

Regarding HLA mismatches, no continuous trend was observed: one mismatch was associated with correct classification more often than two or no mismatches. Similarly, for total HLA mismatches, an intermediate range of three mismatches was linked to better classification, rather than a linear increase or decrease. Visual analysis of HLA mismatch distribution by donor type suggested an association between an intermediate number of mismatches and living donors, indicating an interrelationship between HLA mismatch count and donor type.

### Subgroup analysis of score performance

Based on prior analyses, we investigated whether a clinical subgroup could be identified in which the score reliably differentiates between rejection cases and controls, even within the first 14 days post-transplant. To this end, we evaluated all parameters that were significant in both univariate and multivariate analyses, identifying the subgroup within each parameter category that achieved the best discriminatory performance. Given its particular clinical relevance, eGFR was also included in this subgroup analysis, which was performed by comparing the area under the curve (AUC) of the receiver-operating-characteristic (ROC) curve across subgroups (see Table 3). Total HLA mismatch was excluded due to insufficient case numbers.

**Table 3:** Overview of subgroup specific area under the curves (AUC) of the receiver-operating-characteristic (ROC) curves.

Parameter	Subgroup characteristic	Subgroup AUC (95% CI)
Living donor	yes	0.720 (0.62–0.82)
Warm ischemia time	≤ 30 min	0.702 (0.61–0.79)
Cold ischemia time	<200 min	0.689 (0.59–0.79)
Induction therapy	yes	0.618 (0.55–0.68)
eGFR	≥ 30 mL/min/1.73m^2^	0.601 (0.48–0.72)
Age	≥ 50 years	0.589 (0.53–0.65)
Residual excretion	≥ 500 mL	0.435 (0.27–0.60)

For each parameter that had a significant effect on sample misclassification in the univariate and/or multivariate analyses, a ROC curve was generated showing the discrimination between cases and controls by the urinary metabolite constellation for the best performing subgroup in each case. The results are presented here using the AUC with 95% confidence interval (CI) of the ROC curves for comparison. The AUC for all samples collected during the inpatient phase was 0.52 (95% CI, 0.47–0.57).

**Figure 2:**
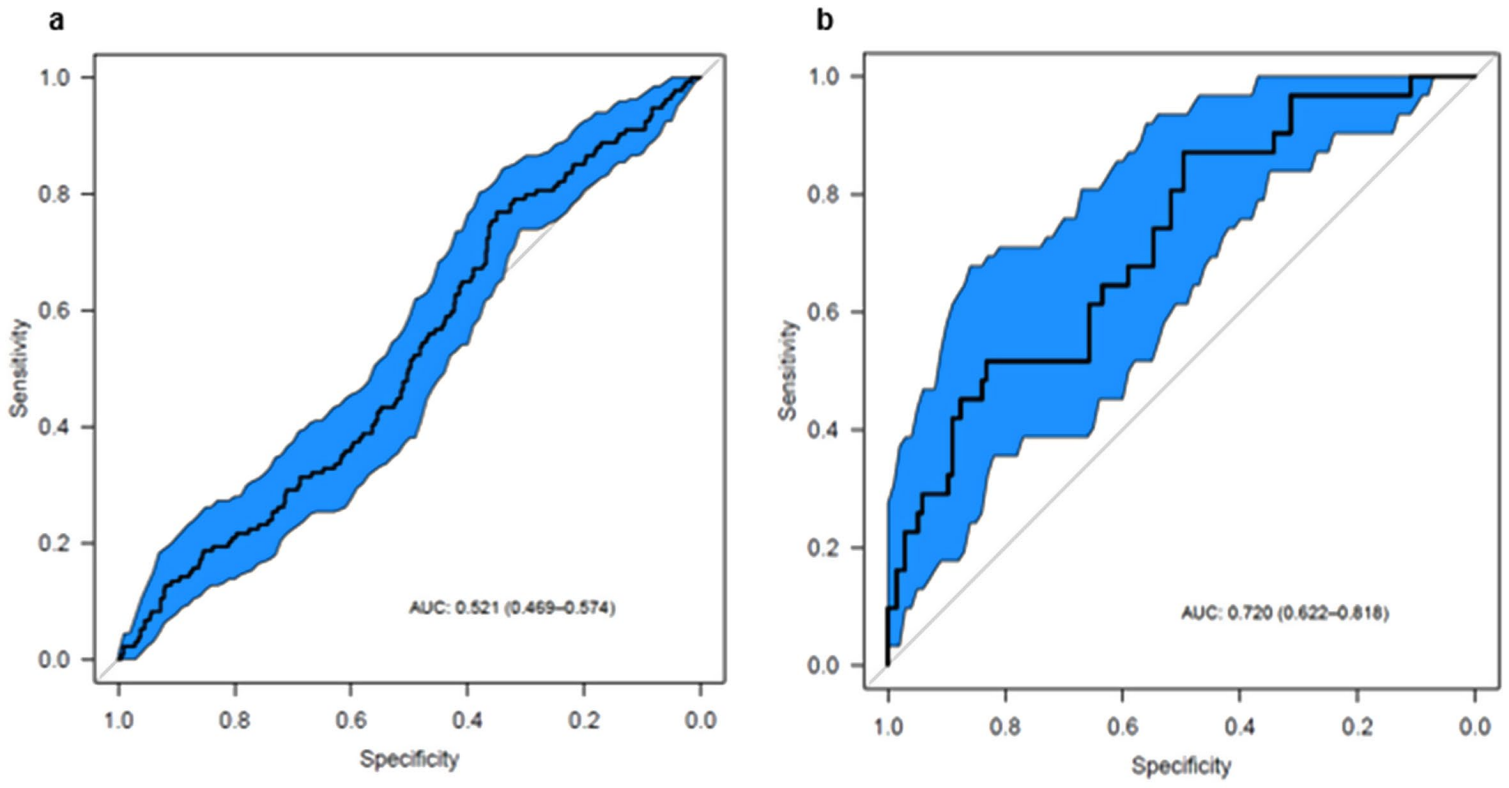
ROC curves for the urinary metabolite rejection score. The fraction of true positive results (sensitivity) and the fraction of false positive results (1–specificity) for the metabolite constellation are shown. Blue area represents upper and lower 95% confidence intervals of the ROC curve. **(a)** Considering all samples from day 1–14 post-transplant with 134 cases and 548 controls, the urinary metabolite constellation is not able to discriminate between renal rejection and no rejection (AUC of 0.52 with 95% CI: 0.47–0.57). **(b)** For patients with a living donor, the test performs with an AUC of 0.72 (95% CI, 0.62–0.82) based on 31 case and 137 control samples. This comes close to the original test performance from the outpatient phase (AUC of 0.75, 95% CI: 0.68–0.83).

The strongest performance was observed in recipients of living donor organs. In this subgroup, the score discriminated between rejection and control patients nearly as effectively as in the initial outpatient cohort (AUC = 0.72; 95% CI: 0.62–0.81 vs. AUC = 0.75; 95% CI: 0.68–0.83; see Figure 2).

The second-best performance was seen in patients with a short warm ischemia time (≤ 30 min), with a slightly lower AUC of 0.70 (95% CI, 0.61–0.79). Similarly, patients with a cold ischemia time ≤ 200 min demonstrated an AUC of 0.69 (95% CI, 0.59–0.79). Notably, in this subgroup, all but one patient received a living donor kidney.

Subgroups defined by induction therapy, estimated glomerular filtration rate (eGFR), and age yielded AUCs ranging between 0.59 and 0.61.

Although residual urine output was the most frequently significant parameter in multivariate analyses, subgroup analysis did not reveal any discriminatory ability between rejection and non-rejection in any residual excretion category (overall AUC = 0.44; 95% CI: 0.27–0.60). Even in the best-performing subgroup—patients with residual urine output >500 mL—only a limited number of cases was available (*n =* 10).

We also assessed various combinations of patient characteristics, but none achieved an AUC above 0.70. The best-performing combination—patients with eGFR ≥ 30 mL/min/1.73 m^2^ and induction therapy—achieved an AUC of 0.70 (95% CI: 0.54–0.86). In comparison, the combination of eGFR ≥ 30 mL/min/1.73 m^2^ and living donation yielded a lower AUC of 0.60 (95% CI: 0.52–0.69).

An analysis of intra-individual score trajectories during the first 20 days post-transplant indicated that false-positive elevations were most commonly associated with low urine output, prolonged catheter use or self-catheterization, perirenal lymphocele formation, voiding dysfunction, or urinary stasis.

## Discussion

This *post-hoc* sub-analysis of the UMBRELLA study focused on the first 14 days after kidney transplantation and examined potential confounding factors that could influence the performance of a previously established urinary metabolite signature used to detect renal allograft rejection. Out of 29 possible confounders, 10 were supported by univariate and multivariate analyses. Notably, post-mortem organ donation was linked to a significantly altered urinary metabolic profile, which could confound the metabolite score. Conversely, in recipients of living donor kidneys, the score was nearly as effective at distinguishing rejection from non-rejection cases within the first 14 days as it was beyond day 15. This is particularly interesting because most earlier biomarker studies excluded the early post-transplant period due to inconsistent results (16–19).

Most of the other significant confounders—such as prolonged cold ischemia time, reduced residual diuresis, absence of induction therapy, lower eGFR, younger recipient age, and specific HLA mismatch patterns—were mainly associated with the donor type, especially differentiating deceased from living donor transplants. Living donor kidneys are transplanted immediately after retrieval, resulting in shorter cold ischemia times, while deceased donor organs often experience longer cold storage, which can stress the graft and impact its function. This stress may alter urinary metabolites, affecting the accuracy of rejection detection (21–25).

On a biochemical level, extended cold ischemia has been shown to induce tissue alkalosis in mouse kidneys (26). Conversely, shorter ischemia times are associated with reduced anaerobic metabolism and less ischemia–reperfusion injury, which may ultimately improve the accuracy of biomarker-based rejection detection. Lower urine output following prolonged ischemia may in turn alter urinary metabolite concentrations.

Residual diuresis is generally higher in recipients of living donor kidneys, who tend to be transplanted earlier in the course of their disease—often preemptively and with shorter durations on dialysis (27). Residual urine output is known to decline with time on dialysis (28), and higher residual diuresis reflects better preserved endogenous renal function and likely less overall systemic impairment. Patients with preserved residual function may thus be less prone to metabolic confounders that mimic rejection. In line with this, patients without residual diuresis experience higher rates of urological complications, such as urinary leakage (28), which may further affect urinary metabolite profiles.

While increased residual diuresis improved classification accuracy in some analyses, subgroup results were inconclusive due to limited sample sizes.

All living donor transplant recipients and several deceased transplant recipients in our cohort received induction therapy, consistent with clinical practice that prioritizes this treatment in patients expected to have longer graft function and favorable risk profiles. Induction therapy may influence immune activation patterns and thereby impact biomarker expression. However, the strong correlation between induction therapy and living donation introduces bias that complicates the interpretation of these individual variables.

Living donor transplants also tend to have better initial graft function and are less affected by ischemia–reperfusion injury, often resulting in higher early eGFR values (27). In contrast, deceased donor grafts—particularly those from older or marginal donors—carry an inherently higher risk of graft failure (29), which may also alter rejection-related biomarker levels.

HLA mismatch patterns further influence immune activation and biomarker expression. Although living donor transplants often involve greater HLA mismatches, they still demonstrate superior outcomes compared to deceased donor transplants with better HLA compatibility (30). This is likely due to donor selection strategies that avoid complete incompatibility, resulting in a moderate level of mismatch.

Since many of the identified confounders are intrinsically linked to donor type, it is crucial to consider this when evaluating early post-transplant biomarkers. Differences in ischemia times, graft function, and immune status between donor types may significantly alter urinary metabolite patterns, associated with rejection, thus affecting diagnostic accuracy. Future biomarker studies should adjust for donor type to ensure reliable biomarker performance across different clinical scenarios.

Our results suggest a considerable metabolic impact of donor status. Whether the graft is from a living or deceased donor significantly influences urinary metabolite patterns. This finding aligns with previous studies that identified deceased donor organs as risk factors for renal tubular acidosis (31) and acute tubular necrosis (27). Additionally, impaired perfusion and anaerobic metabolism have been documented as kidney-specific responses following brain death in animal models (32).

Among the remaining factors, warm ischemia time was also associated with altered urinary metabolite profiles. The subgroup with short warm ischemia time showed the second-best AUC performance after the living donor subgroup. This is consistent with pathophysiological mechanisms involving ischemia–reperfusion injury, which may arise from either prolonged cold or warm ischemia. Both have been linked to graft failure and delayed graft function (33). At the molecular level, hypoxia impairs Na^+^/K^+^-ATPase activity, leading to metabolic dysregulation (34). Prolonged warm ischemia could exacerbate such effects, reinforcing the need to consider this variable in metabolic analyses.

Interestingly, for donor-derived cell-free DNA (dd-cfDNA), both donor type and warm ischemia time have also been shown to influence biomarker levels immediately post-transplant, similar serum creatinine and delayed graft function (35). However, in our cohort, the limited number of patients with delayed graft function (cases: *n =* 8; controls: *n =* 15) precluded meaningful subgroup analysis. While we also examined eGFR as a potential confounder, its effect was clearly secondary to donor status and warm ischemia time.

A key limitation of our subgroup analysis is the small number of samples per subgroup, which limits statistical power, particularly given the low proportion of rejection cases. Univariate and multivariate analyses had to be performed across six different data stratifications (case/control and various cut-offs), and significant confounders were identified primarily in the control settings, with one exception. This may reflect the smaller sample size in the rejection group, which could mask meaningful associations. Alternatively, it may indicate that in the unselected early post-transplant cohort, the score’s performance is primarily limited by false-positive elevations in metabolically perturbed controls mimicking rejection, rather than by an inability to detect rejection. This effect is particularly pronounced in deceased-donor recipients with ischemia-related metabolic alterations. In contrast, in the living-donor subgroup with fewer confounders, the score reliably discriminated rejection from non-rejection, demonstrating that early diagnostic discrimination is feasible under conditions of minimal graft stress.

Moreover, the multivariate analyses were restricted to parameters with at least 30 correctly and incorrectly classified samples to ensure robustness. While this improves reliability, it also excludes potentially meaningful but underrepresented variables, suggesting that some biologically relevant confounders may not have been adequately captured.

In addition, 9 of the 109 patients included in this study underwent simultaneous pancreas–kidney transplantation (SPK). Because urinary metabolites predominantly reflect renal graft function and substantial interference from the pancreatic graft is unlikely, SPK recipients were included in the analysis. However, the SPK subgroup was too small to allow adequately powered comparisons between kidney transplantation alone and SPK. We therefore cannot exclude small transplant type effects.

Upon reviewing the individual cases prospective follow-up indicated that false-positive score elevations were frequently associated with clinical complications such as oliguria, prolonged catheterization, self-catheterization, lymphocele formation, voiding dysfunction, and urinary stasis. Because these variables were not consistently recorded, statistical evaluation of their impact was not feasible. However, it remains plausible that they contribute to metabolic alterations in urine, especially in the early post-transplant period.

Despite these limitations, the study offers important strengths. The performance of the urinary metabolite score was evaluated directly, using a unified model applicable across the dataset without requiring case-specific adjustments. This avoids complications associated with multiple stratifications and enhances the practical utility of the score.

Furthermore, the prospective and longitudinal design of the UMBRELLA study allowed daily urine sampling during the inpatient phase. This enabled analysis of intra-individual score trajectories over time and under varying clinical conditions, providing a rich dataset for future refinement of metabolite-based rejection diagnostics.

## Conclusion

The previously established urinary metabolite signature for the detection of acute renal allograft rejection demonstrated robust diagnostic performance within the first 14 days post-transplantation, but notably only in recipients of living donor kidneys. This observation suggests that distinct metabolic alterations may occur in recipients of deceased donor grafts during the early post-transplant period, potentially limiting the accuracy of biomarker-based diagnostics in this subgroup.

Despite the known challenges of early post-transplant monitoring, this test represents a valuable adjunctive tool for the early detection of rejection in living donor recipients. It enables closer and more precise surveillance from the outset of the post-transplant course.

## Data Availability

The data underlying this study consist of proprietary metabolomics datasets generated by numares AG. Due to company intellectual property policies and the competitive sensitivity of these datasets, the raw data cannot be made publicly available. However, the data can be made available to qualified researchers upon reasonable request. Access will require execution of a confidentiality agreement (non‑disclosure agreement, NDA) to protect company intellectual property. Requests may be directed to the corresponding author or to numares AG, and will be evaluated in accordance with institutional and legal guidelines.
