# Supplementary material for: Diagnosis of early kidney allograft rejection: influencing factors in metabolite-based urine analysis
Source: Front Med (Lausanne). 2026 Mar 6;13:1688235. doi: 10.3389/fmed.2026.1688235 (PMC13002353; doi:10.3389/fmed.2026.1688235)
Supplement: Supplementary file 1 [file Presentation_1.PPTX]

## Slide 1
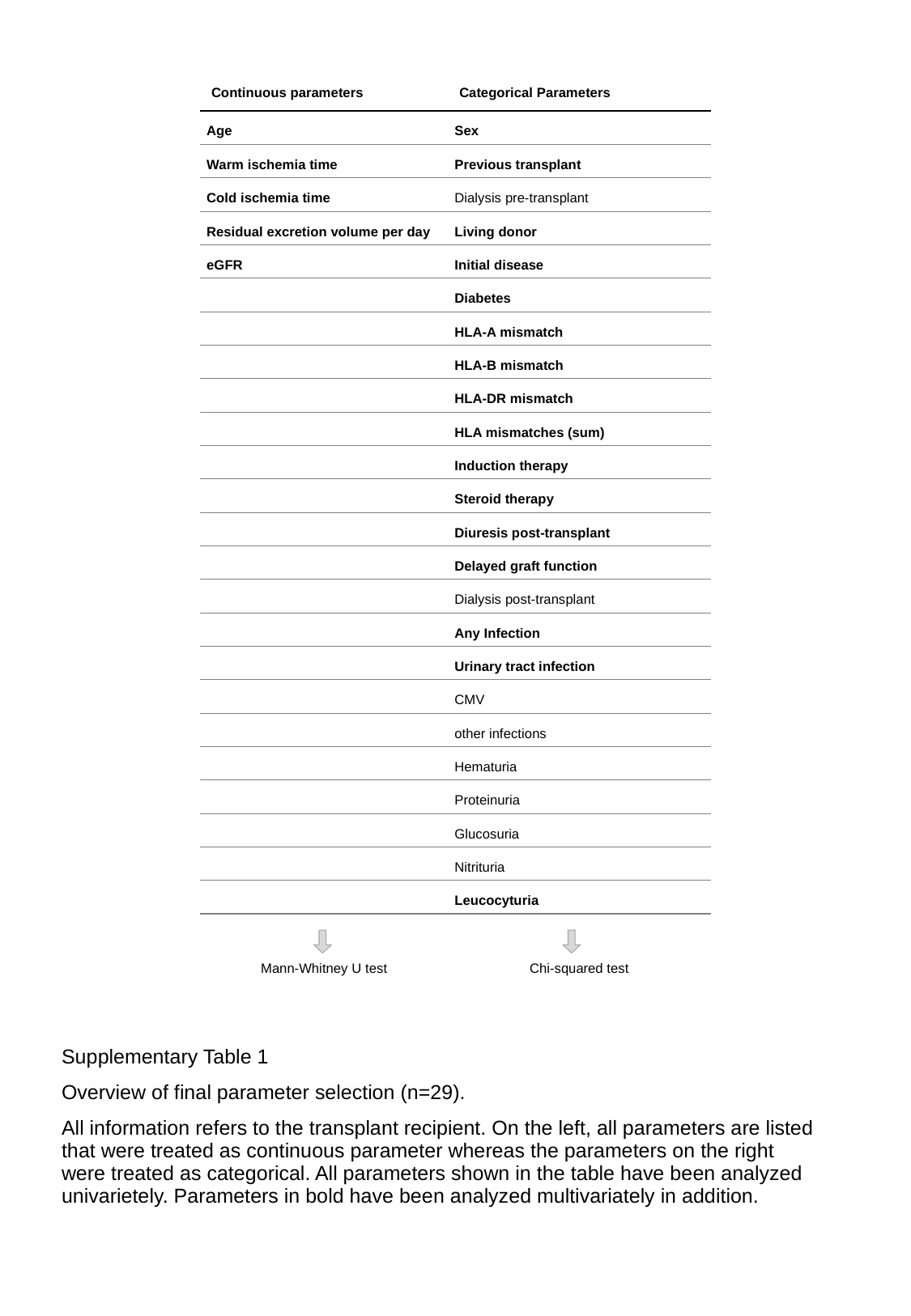

| Continuous parameters | Categorical Parameters |
| --- | --- |
| Age | Sex |
| Warm ischemia time | Previous transplant |
| Cold ischemia time | Dialysis pre-transplant |
| Residual excretion volume per day | Living donor |
| eGFR | Initial disease |
| | Diabetes |
| | HLA-A mismatch |
| | HLA-B mismatch |
| | HLA-DR mismatch |
| | HLA mismatches (sum) |
| | Induction therapy |
| | Steroid therapy |
| | Diuresis post-transplant |
| | Delayed graft function |
| | Dialysis post-transplant |
| | Any Infection |
| | Urinary tract infection |
| | CMV |
| | other infections |
| | Hematuria |
| | Proteinuria |
| | Glucosuria |
| | Nitrituria |
| | Leucocyturia |
| Mann-Whitney U test | Chi-squared test |
Supplementary Table 1
Overview of final parameter selection (n=29).
All information refers to the transplant recipient. On the left, all parameters are listed that were treated as continuous parameter whereas the parameters on the right were treated as categorical. All parameters shown in the table have been analyzed univarietely. Parameters in bold have been analyzed multivariately in addition.

## Slide 2
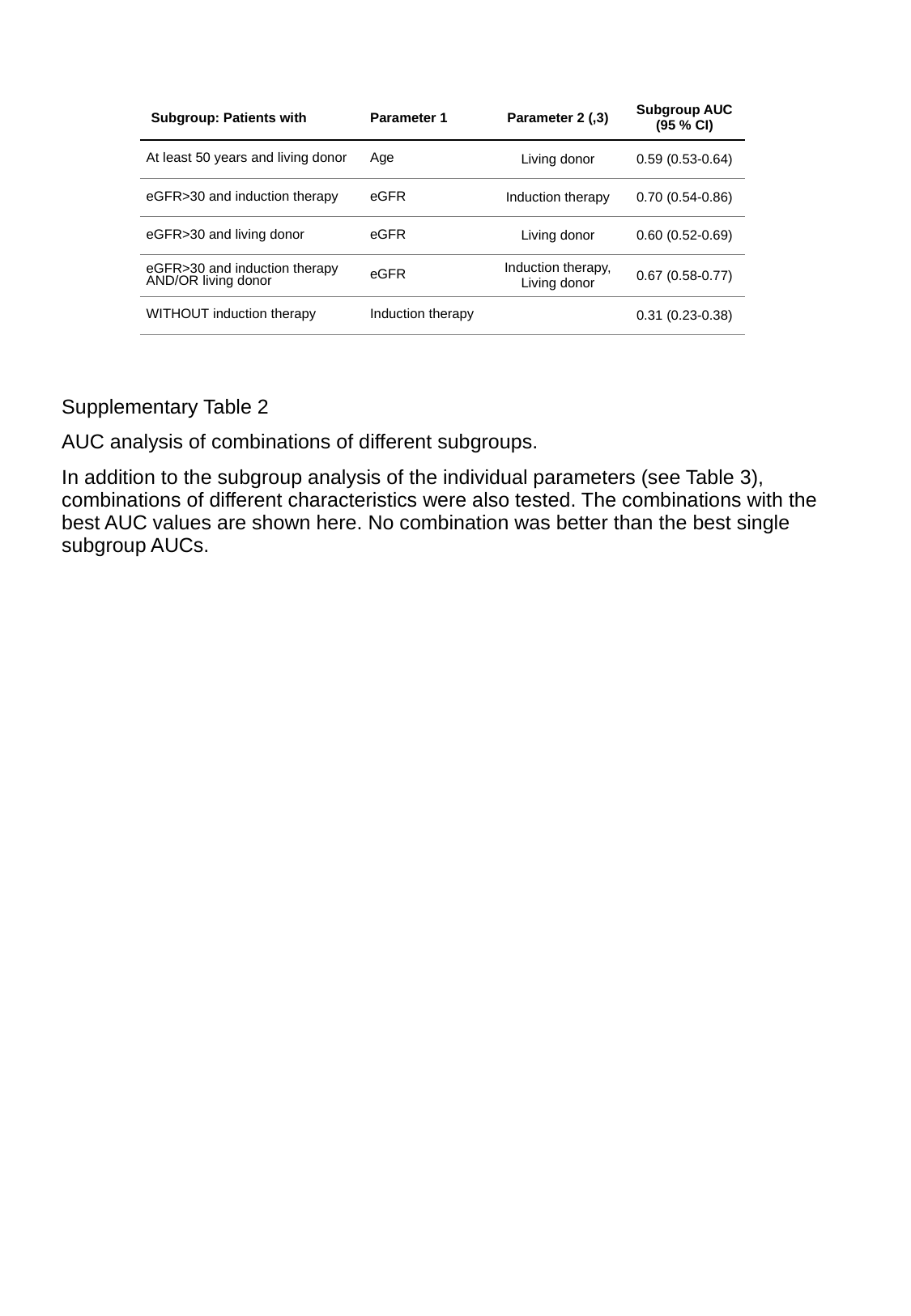

| Subgroup: Patients with | Parameter 1 | Parameter 2 (,3) | Subgroup AUC (95 % CI) |
| --- | --- | --- | --- |
| At least 50 years and living donor | Age | Living donor | 0.59 (0.53-0.64) |
| eGFR>30 and induction therapy | eGFR | Induction therapy | 0.70 (0.54-0.86) |
| eGFR>30 and living donor | eGFR | Living donor | 0.60 (0.52-0.69) |
| eGFR>30 and induction therapy AND/OR living donor | eGFR | Induction therapy, Living donor | 0.67 (0.58-0.77) |
| WITHOUT induction therapy | Induction therapy | | 0.31 (0.23-0.38) |
Supplementary Table 2
AUC analysis of combinations of different subgroups.
In addition to the subgroup analysis of the individual parameters (see Table 3), combinations of different characteristics were also tested. The combinations with the best AUC values are shown here. No combination was better than the best single subgroup AUCs.
